# The macroeconomic effects of COVID-19 in Montenegro: a Bayesian VARX approach

**DOI:** 10.1186/s40854-020-00207-z

**Published:** 2020-11-10

**Authors:** Gordana Djurovic, Vasilije Djurovic, Martin M. Bojaj

**Affiliations:** Faculty of Economics, University of Montenegro, Boulevard Jovana Tomasevica 37, 81000 Podgorica, Montenegro

**Keywords:** Pandemics, Risk, Macroeconometrics, Forecast

## Abstract

This study examines, diagnoses, and assesses appropriate macroeconomic policy responses of the Montenegrin Government to the outbreak of COVID-19. The model econometrically measures the macroeconomic costs using a Bayesian VARX Litterman/Minessota prior to the pandemic disease in terms of demand and supply loss due to illness and closed activities and their effects on GDP growth in various pandemic scenarios. We explore five economic scenarios—shocks—using the available data from January 2006 to December 2019, following real out-of-sample forecasts generated from January 2020 to December 2020. Sensitivity scenarios spanning January 2020 to June 2020 from ± 10 to ± 60% were analyzed. We observed what happens to the supply and demand sides, namely, GDP, tourism, capital stock, human capital, health expenditures, economic freedom, and unemployment. The results show a toll on the GDP, tourism, unemployment, capital stock, and especially human capital for 2020. The recommended policy measures are public finance spending initiatives focused on securing employment and keeping highly qualified staff in Montenegrin companies. Considering all uncertainties, the rebound of the Montenegrin economy could take a few years to reach pre-COVID 19 output levels.

## Introduction

In late December 2019, a cluster of unexplained pneumonia cases were reported in Wuhan, China. A few days later, the causative agent of this mysterious pneumonia was identified as a novel coronavirus. The World Health Organization (WHO) named this causative virus severe acute respiratory syndrome coronavirus 2 and the associated infectious disease coronavirus disease 2019 (COVID-19) (He et al. 2020). Developing at an incredible speed, the health crisis has grown into a deep economic crisis that is affecting the entire world.

The pandemic disease COVID-19, as we write, continues to spread across the integrated world in the absence of a vaccine to prevent outbreaks. No health system in the world is capable of effectively controlling the patient load. The epidemic seems to have initiated in China, and after four weeks, other epicenters were identified, such as Japan, Iran, Italy, Spain, Germany, the United Kingdom, and the United States. There is continuing uncertainty about the emergence of new cases and locations.

Each country is facing economic consequences. Policymakers are trying to identify concrete measures to counter the economic impacts of the COVID-19 outbreak. Stock markets have been dropping drastically. The Dow Jones Industrial Index fell by 36.4% between February 18, 2020 and March 23, 2020 (DJIA 2020). The US Federal Reserve has taken extraordinary measures to support market functioning and economic resilience. Most countries went the same route and created generous support measures for businesses and citizens.

The focus of this study is the analysis of the effect of the health crisis COVID-19 on the economy of Montenegro, which is a small, open, euro-ized economy reliant on tourism situated in southeast Europe. Montenegro’s gross added value is distributed as follows: 8.2% in agriculture, 12.5% in industry (including energy production), 7.2% in construction, and 72% in services, of which direct tourism accounts for around 10%. Employment in the service sector is 82%. The current account deficit is 15% of GDP. The GDP/public debt ratio was 77% in 2019. The country’s maneuverability for new borrowing is limited considering its annual debt service obligations are 11% of GDP (Central Bank of Montenegro 2020). The growth model is based on steady foreign direct investment inflow and services. The main foreign policy strategic priority of this Western Balkans country is full-fledged membership to the European Union. The COVID-19 pandemic has significantly affected Montenegrin economy and livelihoods and will create a recession in 2020 (instead of a 3.4% expected real growth rate).

The economic growth projections range from − 1.3% (World Bank, February 2020) to − 3% (Central Bank of Montenegro 2020) and down to − 9% (IMF, April 2020). The full extent of the impact is difficult to quantify given the evolving nature of the pandemic, but job losses and an increase in poverty are expected.

A limited number of studies have examined the potential consequences of such a pandemic on the economy of Montenegro. The objective of this study is to fill this gap by examining the macroeconomic effects of COVID-19 through analyzing monthly data from January 2006 until December 2019 and out-of-sample data from January 2020 until December 2020 to develop predictive ability regarding the movement of macro model variables. The novelty of the research is that we measure and add the effects of the demand and supply variables cumulatively and then average them. We measure COVID-19’s impact on the Montenegrin economy using a Bayesian vector autoregressive (VAR) and forecasting sensitivity deterministic—dynamic scenario model. We apply alternative forecasting scenarios to five macroeconomic variables. As the pandemic is an exogenous shock, we change the assumptions of the endogenous variables by a certain amount, subject them to a shock, and then observe how the forecast changes as a result of the change in the assumption. Tourism, capital stock, and human capital are dropped hypothetically from − 10%, − 20%, − 30%, − 40%, − 50%, and − 60% from January 2020 to June 2020. Health expenditures and unemployment are increased hypothetically from + 10%, + 20%, + 30%, + 40%, + 50%, and + 60% from January 2020 to June 2020. We assume that the direct pandemic disease lasts 3 months, from January 2020 until March 2020. The side effects and consequences remain for the upcoming 3 months, from April 2020 until June 2020. The assumption is based on the premise that even after the pandemic disease disappears after 3 months, its economic impact continues to deepen the consequences. It is a one-off counter windfall because the pandemic time-horizon from the start to the end is predicted to last for 6 months. That is, we assume that the direct impact starts in January and lasts until March, and the consequent effects last from April until the end of June (6-month scenario with 2, 3-month periods: strong pandemic crisis impact for 3 months and gradual recovery for the subsequent 3 months and 3-month period of gradual recovery). Then, the question is, what impact would that have on the growth of gross domestic gap (GDP_GAP) of Montenegro potentially?

Monthly data give us a more suitable observation period than quarterly or annual data, as the pandemic is evolving and impacting even on hourly and daily bases. Monthly data enable us to observe the transitions from 1 month to the other in the short and long runs. Moreover, the assumptions made in the study include all stages of severity, starting from a low-middle-severe stage. We included all stages because significant uncertainties exist about the pandemic, and different severities help us observe a more comprehensive picture.

The paper is organized as follows. First, we summarize the literature on the economic effects of epidemics. Second, we apply vector autoregression (VARX) and Bayesian vector autoregression (BVARX) under the assumptions of demand and supply shocks and with deterministic—dynamic simulations to study pandemic scenarios. We start with the *baseline* scenario and then modify the macro model by introducing new premises. Third, we present results. Finally, we conclude the manuscript with appropriate suggestions to the central government.

## Literature review

The history of pandemic diseases has led to many macroeconomic studies and methodologies. The most fearsome outbreaks have been the Black Death in the fourteenth century and the Spanish influenza in 1918–1919 (Jonung and Roeger 2006). In the last century, three severe pandemics have influenced the globe (Kilbourne 2006).

Over the past four decades, uncertainty and financial shocks have been examined by many researchers, who have found that these exogenous shocks have played a significant role in business cycle fluctuations (Kou et al. 2014; Caldara et al. 2016; Cesa-Bianchi et al. 2018; Ludvigson et al. 2018; Chao et al. 2019; Kou et al. 2019: Wen et al. 2019; Shen et al. 2020; Wang et al. 2020).

Many studies have linked health and growth (Bhargava et al. 2001; Robalino et al. 2002a, b; WHO Commission on Macroeconomics and Health 2001; Haacker 2004). Still, there is no unanimous consensus among macroeconomists about the recommended methodology and the expected results (Bell and Lewis 2004).

The conventional approach is not appropriate, as it uses only mortality and morbidity as independent variables in estimating the reduction of growth. The chain effect of a pandemic disease is multidimensional, manifesting through labor supply, foreign direct investments, and demand and increasing government expenditure on health care and other activities. Several macroeconomic models have been applied to study the impact of diseases. For example, the G-Cubed multi-country model is a dynamic—stochastic general equilibrium model developed by McKibbin and Triggs (2018) and McKibbin and Fernando (2020). Keogh-Brow et al. (2009) used the COMPACT model on epidemiological data of previous UK influenza pandemics and showed three scenarios of loss: (a) low severity, a loss of 0.58% and 3.35%, yearly and quarterly, respectively; (b) mild severity, a decline of 4.5% and 21%, yearly and quarterly, respectively; and (c) high severity, a loss of 6% and 29.5%, yearly and quarterly, respectively. McKibbin and Fernando (2020) concluded that the scale of costs could be reduced by investing in public health systems in less developed economies. A conventional influenza epidemic panel regression analysis of 43 countries revealed an economic and consumption decline of 6% and 8%, respectively (Barro et al. 2020). Weng (2016) used GDP and consumption growth rates as dependent variables and flu death rates and their 1st and 2nd lags as independent variables to examine the hypothetical potential impact of the Spanish influenza during the 2008–2009 global recession. Their analysis predicted an economic dislocation.

Brainerd and Siegler (2003) proposed that the 1918–1919 pandemic increased economic growth a year later. A similar argument was provided for South Africa (Young 2004). Some researchers have also assessed the model used by the US Congressional Budget Office (2005). All models have two features in common: (a) the consideration of the pandemic from a health perspective and (b) a macroeconomic model.

## Methodology

Even though making predictions ex-ante based on forecasting rather than real results has high macro-econometric uncertainty, macroeconomists still place value on such examinations. The effects of a pandemic result in health expenditures, travel cancelations, and other significant events and changes, which contribute to a depressed economy worldwide (Stojkoski et al. 2020).

Because the Government of Montenegro began implementing the 2030 Agenda on Sustainable Development after adopting the National Strategy for Sustainable Development in 2016, we remodeled our econometric model and the production function as follows (Djurovic et al. 2018):

1 $$\begin{aligned} GDP\_GAP_{t} = & \beta_{0} + \beta_{1} CapitalStock_{t} + \beta_{2} Unemployment_{t} \\ & \quad + \beta_{3} logTourism_{t} + \beta_{4} logHumanCapital_{t} \\ & \quad + \beta_{5} healthexp_{t} + \beta_{6} logeconomicfreedom_{t} + u_{t} , \\ \end{aligned}$$

where $GDP\_GAP_{t}$ denotes the gross domestic growth-HP filtered gap, $CapitalStock_{t}$ denotes the gross fixed capital formation (% of GDP), $unemployment_{t}$ denotes the unemployment rate, $logTourism_{t}$ denotes the natural logarithm of international tourism (number of arrivals), $logHumanCapital_{t}$ denotes the natural logarithm of human capital (in this case, this series consists of those employed and possessing higher education, which is critical for smart and sustainable growth), $healthexp_{t}$ denotes the Health Insurance Fund expenditures to GDP ratio, and $logeconomicfreedom_{t}$ denotes the natural logarithm of economic freedom. The data are sourced from the World Bank, except for: human capital time series sourced from the Statistical Office of Montenegro (Monstat 2020), health expenditure sourced from the Central Bank of Montenegro (CBCG 2020), and economic freedom sourced from the Heritage Foundation (Index of Economic Freedom 2020). The time series have been interpolated to monthly data and seasonally adjusted. Because COVID-19 poses a symmetric shock to the economy, the gross fixed capital formation, tourism, health expenditures, and economic freedom summarize impacts on the demand side (linked to the FDI driven model of economic growth). In contrast, human capital and unemployment are related to effects on the supply side. We employ a New Keynesian macro-model, where GDP growth is modeled with a neoclassical production function using capital and labor as input (Roeger and Veld 2004). We use the output gap because as Giordani (2004) pointed out, using the level of output makes little sense, as using the output gap in the structural vector autoregression (SVAR) substantially reduces the complexity. We consider unemployment and human capital on the supply side, as the impact of the disease incapacitates a portion of the employment force as well as limits those who care for the incapacitated ones. We use capital stock, tourism, health expenditure, and economic freedom to measure the demand side. According to estimates by the World Tourism and Travel Organization (WTTO), the contribution of the tourism and travel sector to the overall Montenegrin economy in 2019 was 31.2% of GDP, and the real GDP growth rate of tourism and travel in the same year was 6.1%, which was almost twice the country’s GDP growth rate. Directly and indirectly, the sector creates 66,900 jobs, which is about a third of the total registered employment (WTTO 2020). In this backdrop, the Government of Montenegro has taken an appropriate national strategy to respond to the COVID-19 pandemic (Government of Montenegro 2020).

First, analysis of the GDP_GAP pandemic influenza determinants are not prevailing, combining theory and empirical results. Second, we use Bayesian VARX to identify a recursive structural model, and we sum up all the effects of each scenario to the corresponding variable, followed by averaging the cumulative effect of the five scenarios. In the context of the Montenegrin economy, which is a small open economy where a variable can be considered strictly exogenous while determining the formulation of the VAR, we add the Serbian output gap. The intuition behind this is that the foreign variable would be expected to impact the domestic economy. It imposes zero restrictions on the $B_{j}$ in our VAR and leads to the VARX model (the X denoting VAR with the exogenous variable).

The aforementioned methodology has not yet been applied to Montenegrin data. As policymakers are interested in hypothetically seeing the trend of the GDP in different situations, we apply different scenarios, such as a decrease in tourism, human capital, and capital stock and an increase in health expenditures and unemployment. We design hypothetical shocks that each simultaneously affect one another. We sum up the effects to have a better view of the cumulative macroeconomic reaction of economic growth to COVID-19.

### Empirical results

Based on unit root tests of the ADF, PP, and KPSS stationary tests, the variables have stationarity. Visual inspection along with correlograms confirms the stationarity. Structural breaks are identified using stability diagnostics, such as recursive estimates, Chow breakpoint test, Quandt-Andrews, and Bai-Perron.

Recursively, we identify a SVARX model of GDP. All the criteria suggest a fitting length of 2 lag orders, as indicated by Clark and Ravazzolo (2015). The stationarity of the VAR (2) is confirmed, as all the inverse roots of the characteristic polynomial lie within the unit circle, as seen in Fig. 5 (in “Appendix”).

Table 2 (in “Appendix”) shows the VARX (2) based on the autocorrelation LM test for serial correlation. The null hypothesis of no serial autocorrelation is accepted up to order 4, which is confirmed by the correlogram residual test that shows no sinusoidal waves.

We are interested in forecasting the economic upheaval due to COVID-19, which appears to be more severe than what the globe experienced during the financial crisis of 2008–2009.

We note that there are two ways of forecasting. The first is within the sample, which cuts the sample into two parts. The first part of the sample is used to make an estimation, and the second part of the data that have not been included in the regression is used for forecasting. The other option is to use all of the data available and do out-of-sample forecasting. To generate a forecast, we can use known values or forecasted values. Using the known values for forecasting is called static forecasting, where we go back to the original data and ignore the previous forecast and use the actual value to generate the forecast. This technique ignores any forecasting errors. In case we use the forecasted values from the regression, it is referred to as dynamic forecasting.

In our case, we use static forecasting using the known values, as the government does not want forecasted errors to increment on themselves. The government adjusts the operations and interventions as needed; therefore, we use static forecasting. We use deterministic simulation, where we obtain only one value for the solution, which does not respond to shocks (yielding a single forecast rather than a distribution of possible values). It calculates under the current set of assumptions or known facts without any shocks introduced, which is called the baseline.

**Fig. 1:**
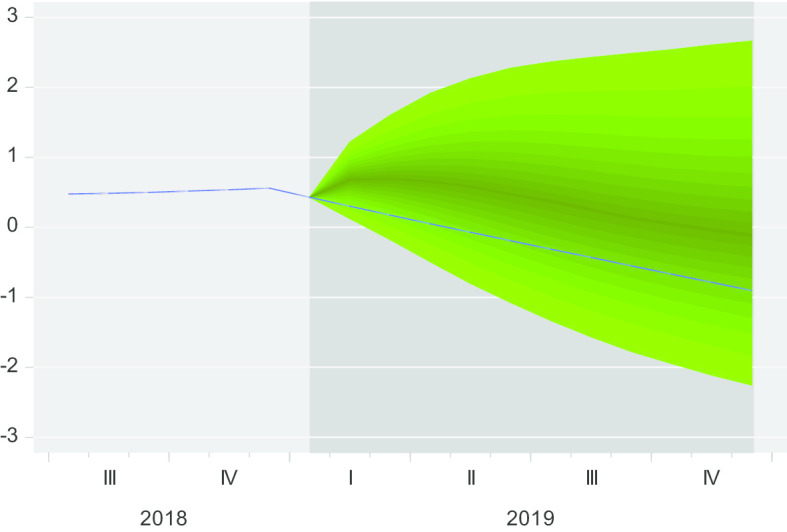
Fan chart forecasting performance of VAR (2). *Source*: Authors' calculations

As can be noticed in Fig. 1, the GDP model fits best into a 45% confidence band throughout the year for the forecasting performance, confirming that the model is well-fitted for prediction. In this case, in order to see how well the model can predict future performance, we estimate a subsample from 2006M01 to 2018M12 and perform forecasting. The results from Fig. 1 imply that we can move further and make real out-of-sample predictions for the time span 2020M01 until 2020M12.

Let us see what a Bayesian VAR model tells us about the probability of a COVID-19 impact-shock on the Montenegrin economy. Using Bayes’ theorem, we can calculate this probability. A regression model with unknown coefficient $\beta$, the variance–covariance matrix $\sum_{e}$, and $e_{t \sim } nid N\left( {0, \sum_{e} } \right)$ along with Bayes’ theorem is employed to combine the prior distribution of the parameters with the likelihood function of the data to produce a posterior distribution of the coefficients $\beta$ (Ouliaris et al. 2018; Chin and Li 2018):

2 $$\overbrace {{p\left( {\beta |\sum_{e} , Y} \right)}}^{posterior} = \frac{{ \overbrace {{L\left( {Y|\beta , \sum_{e} } \right)}}^{likelihood}\;\overbrace {{p\left( {\beta |\sum_{e} } \right)}}^{prior} }}{p\left( Y \right)},$$

where $p(Y) = \smallint p\left( {Y|\beta } \right) p\left( \beta \right)d\left( \beta \right)$ is a normalizing constant. The posterior distribution is the likelihood function times the prior distribution:

3 $$p\left( {\beta |\sum_{e} , Y} \right) \propto L\left( {Y|\beta , \sum_{e} } \right) p\left( {\beta |\sum_{e} } \right).$$

In our case, the simplification would look like $p\left( {MNE|shock} \right) = \frac{{L\left( {shock|MNE} \right) p\left( {MNE} \right)}}{{p\left( {shock} \right)}}$, where $p\left( {MNE|shock} \right)$ denotes the impact of the innovation to Montenegro, $L\left( {shock|MNE} \right)$ denotes the likelihood ratio (the probability of “MNE” being true given “shock” is true), $p\left( {MNE} \right)$ denotes the probability of “MNE” being true, and $p\left( {shock} \right)$ denotes the marginalization (the probability of “shock” being true). Bayes’ theorem allows us to update our opinion based on new information. Previous posterior beliefs (updated opinion) are today’s prior (opinion to be updated). The idea about updating beliefs is core to Bayesian econometrics and can be used to test the hypothesis. We start with some idea or opinion based on econometric inference about how something works. Simulation methods of different types of priors get us different posteriors. In the case of the normal distribution of the prior $\beta$, the normal distribution will have the posterior as well, and the matrix weighted average of the OLS estimates of the mode and mean of the prior $\beta$ are

4 $$\overline{b} = \left[ { \underline{{V^{ - 1} }} + \sum_{e}^{ - 1} \otimes \left( {X^{\prime } X} \right)} \right]^{ - 1} \left[ { V^{ - 1} \underline {b} + \left( {\sum_{e}^{ - 1} \otimes X^{\prime } } \right)y} \right].$$

As seen from the above expression (4), Bayesian methods tend to shrink the VAR-estimated coefficients toward the prior mean and away from the OLS estimates. Forecasting gains are exactly this just-mentioned characteristic: shrinking the VAR estimates toward the prior mean. VARs often end up with the overfitting problem, which can result in imprecise forecasts. As VARs have many parameters to estimate *n*(*np* + 1), often inaccurately because of limited data, consequently, the response functions and forecasts are not well-determined. Thus, the number of coefficients easily proliferates. Standard error bands tend to not account for parameter uncertainty, making forecasts to look more precisely than they are really. The Bayesian method introduces prior distributions, including parameter uncertainty. The idea is to have a parsimonious model and a restricted number of parameters being estimated. The literature contains practical solutions to omit some lagged values *p* in some equations, which is usually referred to as “best sub-set VARs.” In this section, we apply Bayesian methods, which set valuable prior distributions on the whole structure of the VAR coefficients to obtain a parsimonious model.

Relative to our previous VAR model, we estimate the BVAR prior type of Litterman/Minnesota, Normal-Flat, Independent Normal-Wishart, Sims-Zha (normal-Wishart), and Giannone–Lenza–Primiceri to perform out-of-sample forecasting from January 2019 to December 2019. Our primary variable of interest is the GDP_GAP. Table 1 shows the results. There is an improvement in the forecasts.

**Table 1:** Forecasting using Bayesian estimation prior types 2019:1–2019:12. *Source*: Authors' calculations

Prior	Variable	RMSE,$\lambda_{1} = 0.5$	RMSE,$\lambda_{1} = 1.0$
Standard VAR	GDP_GAP	0.149639	0.149639
Minnesota	GDP_GAP	**0.059793**	0.068993
Normal-Flat	GDP_GAP	0.066566	0.066566
Normal-Wishart	GDP_GAP	0.066562	0.066562
Sims-Zha (N-W)	GDP_GAP	0.062344	0.071688
Giannone, L & P	GDP_GAP	0.063592	**0.063770**

Bold values are the lowest RMSE

Compared to the standard VAR (2) estimate, the BVARX of Litterman/Minnesota prior has the lowest root mean square error (RMSE) of 0.059793 given the setting $\mu_{1} = 0$. As we have proven that the VAR (2) is stationary and $\lambda_{1} = 0.5$, it implies a relatively uncertain prior for $\beta$.

Standard deviations of the first variables in each equation are controlled through $\lambda_{1}$ and shrinking the first-lag coefficients. Relative to the VAR parameter estimates, the lagged coefficients have shrunk. The importance of the lagged variables in the *i*-th equation is controlled with lambda2 ($\lambda_{2} = 0.99)$. Lambda3 ($\lambda_{3} = 1)$ determines the lag decay rate through $l^{\lambda 3}$, where *l* is the lag index. We have set $\lambda_{3} = 1$ for no decay. For $\lambda_{1} = 1.0$, the Giannone, Lenza, and Primiceri prior has the lowest RMSE = 0.063770.

**Fig. 2:**
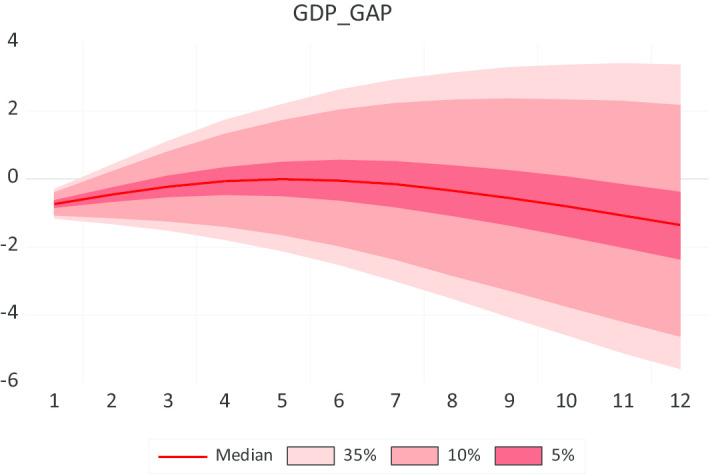
Forecasting performance of GDP_GAP using Bayesian VARX, Litterman/Minnesota prior type, 2020:1 2020:12. *Source*: Authors' calculations

The dark red line represents the median value. The rest represents the fans of the quantiles. Visual inspection reveals that the Bayesian VAR (2) model in Fig. 2 forecasts the best output gap for the time span from January 2020 to December 2020. It shows a forecasting performance of − 1.3% of the output gap on December 31, 2020.

Thus, we have identified the most suitable model for forecasting GDP, namely, the Bayesian VARX of Litterman/Minnesota prior. We must keep in mind that this is the forecasting performance employing the exogenous variable of the Serbian output gap in our Bayesian VARX.

**Fig. 3:**
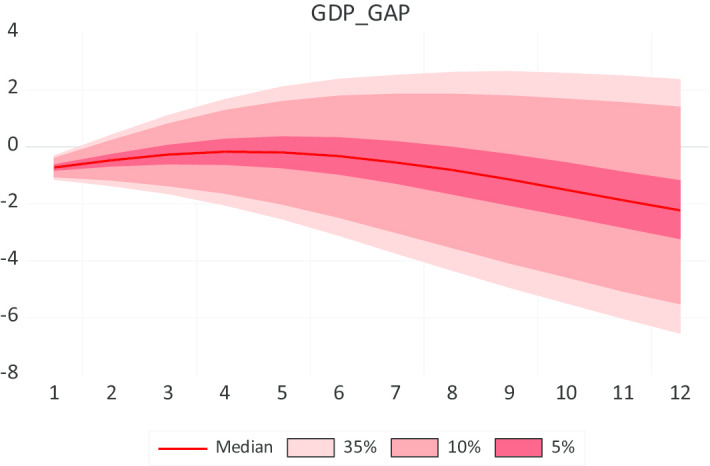
Forecasting performance of GDP_GAP using Bayesian VARX, Sims-Zha (normal-Wishart) prior type, 2020:1 2020:12. *Source*: Authors' calculations

As seen in Fig. 3, the forecasting performance of the GDP_GAP is predicted at − 2.2% using the exogenous variable and the Serbian output gap. It uses a Bayesian VARX Sims-Zha (normal-Wishart) prior type for the period January 2020 to December 2020. Central macroeconomic policymakers are interested in the prediction after adding more exogenous shocks to the model.

### Sensitivity analysis

The macroeconomic policymakers of Montenegro, especially the Ministry of Finance and the Central Bank of Montenegro, are interested in having a close prediction of the GDP following the outbreak of COVID-19. We consider GDP as a leading indicator. The alternative scenarios start altering the data from January 2020 until July 2020. We assume that tourism, capital stock, and human capital decrease from − 10%, − 20%, − 30%, − 40%, − 50%, and − 60%, and health expenses and unemployment increase from + 10%, + 20%, + 30%, + 40%, + 50%, and + 60% from January 2020 to June 2020. We assume that the direct impact occurs from January until March, and from April until the end of June, the economy keeps decreasing, as small and medium businesses deal with a struggle in the first quarter during the recovery in the second quarter. We assume that the impact of the pandemic outbreak ceases at the end of June.

**Fig. 4:**
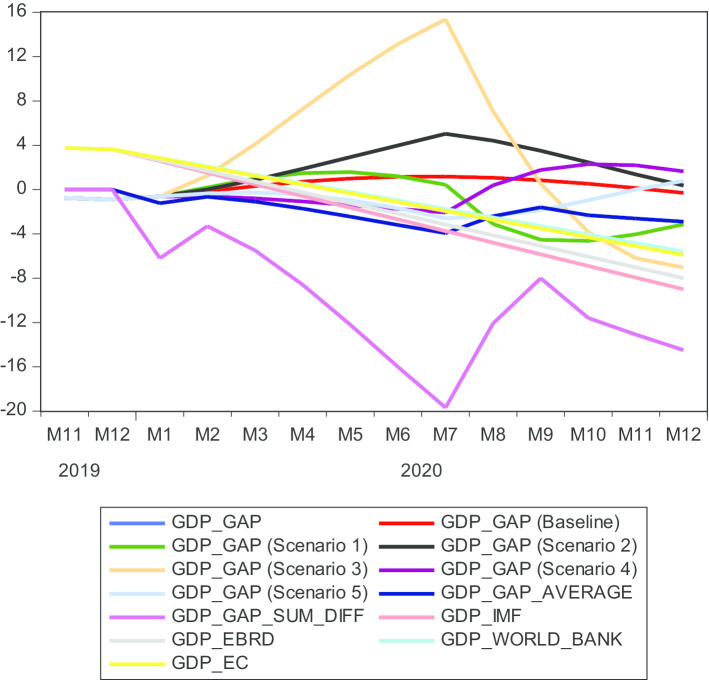
Bayesian VARX Litterman/Minnesota prior type forecasting GDP_GAP and forecast comparison, 2020:1–2020:12. *Source*: Authors' calculations

**Fig. 5:**
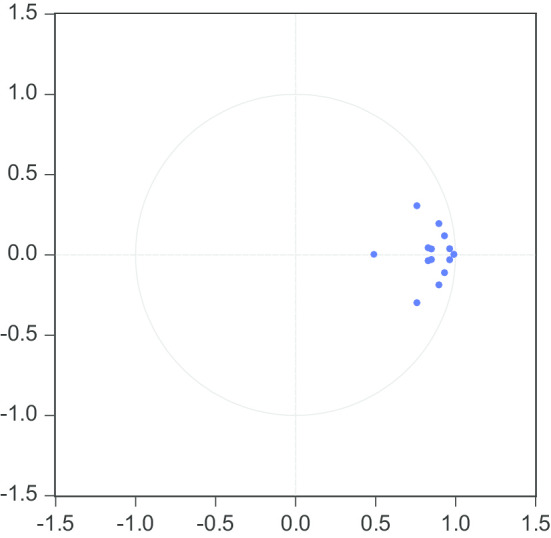
Inverse roots of AR characteristic polynomial. *Source*: Authors' calculations

Scenario 1 represents the international tourism decrease, scenario 2 represents the capital stock decrease, scenario 3 represents the human capital decrease, scenario 4 shows the health expenditure increase, and scenario 5 shows the unemployment increase. In Fig. 4, the blue line denotes the real GDP_GAP, the red line denotes the baseline, the green line denotes scenario 1 (decrease of tourism), the black line denotes scenario 2 (capital stock decrease), the orange line denotes scenario 3 (human capital decrease), the purple line denotes scenario 4 (health expenditure decrease), the light blue line denotes scenario 5 (unemployment decrease), the dark blue line denotes the average of scenarios 1–5 (GDP_GAP_AVERAGE), the violet line denotes the cumulative effect of the scenarios 1–5 (GDP_GAP_SUM_DIFF), the light red line denotes the IMF GDP growth rate projection (GDP_IMF), the light gray line denotes the EBRD GDP growth rate projection (GDP_EBRD), the light green line denotes the World Bank GDP growth rate projection (GDP_WORLD_BANK), and the yellow line denotes the European Commission GDP growth rate projection (GDP_EC).

The dynamic solution does not use actual data; instead, it uses forecasted lagged values over the forecast period. Visual inspection of Fig. 3 reveals that scenarios 1 and 3 (the decrease of international tourism and human capital, respectively) impact the GDP_GAP the most in Montenegro, forecasting a reduction of the GDP_GAP to − 3.2% and − 7.0%, respectively, from January 2020 to December 2020. The recovery of FDI inflow into Montenegro’s economy could be expected soon after the end of the health crisis, as predicted by scenario 2. This is good news for stabilizing the expectations of foreign investors. Why would scenario 2 drag this increase? As Montenegro is in the process of converging toward the European Union, investors (FDIs and their substantial impact on the gross fixed capital formation) would not drop their foreign direct investments. They will resume investing in the Montenegrin economy soon after the COVID-19 pandemic and continue to contribute to increasing the GDP. This is a very promising trend in our findings.

Interestingly, we notice that the GDP_GAP is predicted by scenario 3 to drop sharply to − 7% at the end of December 2020. In other words, as companies let valuable employees leave in the first two quarters of 2020, the multiplicative effect will cause a decrease in the GDP_GAP. The logical conclusion regarding priority government measures is that they should focus on securing employment through different economic-social means during the COVID-19 pandemic. Soon after recovery, the economy will respond positively, and workers will be essential immediately to drive the engine of economic recovery. Therefore, workers should be advised that despite the upheaval caused by the coronavirus pandemic, their jobs are secure. As the supply shocks, through employment and human capital, trigger changes in aggregate demand that are much larger than the shocks themselves, policymakers should take care to not allow firms to exit and cause job destruction (Veronica et al. 2020). The chain effect would cause a recession in the absence of interventions through conventional and non-conventional policies. Unconventional policies are needed because some sectors are shut down, and the traditional fiscal stimulus might not bring them multiplier effects as usual. Shutting down direct human-to-human intensive sectors and giving full insurance payments to the affected workers can achieve the best allocation even though this intervention has a lower per-euro potency of fiscal policy. Large-scale support and stimulus packages should be the primary focus, which is confirmed by the Bayesian VARX Litterman/Minessota prior performance. The fact that human capital is predicted to drop 4.85% only from the capital stock innovations and 2.28% from the tourism innovations informs the policymakers that they should create such policies to keep jobs active during the COVID-19 pandemic. In other words, the government should act as macro-prudentially as possible. Today, governments all over the world are facing a surge in unemployment and implementing measures in support of consumers, small businesses, and corporations.

Health expenditure in scenario 4 predicts the output gap at 1.6% in December 2020, implying that investing in medical equipment will help cure people, bring them back to work, and consequently increase the output gap in Montenegro. Unemployment in scenario 5 decreases the output gap in the first half of the year; from July 2020 to December 2020, it increases the GDP_GAP. That is, those unemployed during COVID-19 will return to their jobs, thus increasing the GDP_GAP. Adding all the impacts of scenarios 1–5 to the output gap, we notice the decrease of the GDP_GAP to − 6.2% in January 2020. Still, in February, the cumulative effect increases the GDP_GAP and then decreases it to − 19.7% in July 2020. After the end of the cumulative effect of the scenarios, the output gap increases to − 8.0% until September 2020. After the hypothetical scenario ends, the economy starts to accelerate from July 2020 to September 2020. Later, the accelerated growth subsides, and it starts decreasing from September 2020 to December 2020, reaching a cumulative negative impact of − 14.5%. We have to keep in mind that the dynamic solution uses forecasted lagged values that cumulate the forecast error from 1 month to the other.

Averaging the scenarios, we notice the dark blue line follows the sinusoidal wave movement of the unemployment scenario 5, health expenditure scenario 4, and international tourism scenario 1 most of the time. In the first quarter, the average output gap drops to − 1.1% and continues decreasing in the second quarter till − 3.2%. At the end of July 2020, it forecasts − 3.9%. After the hypothetical sensitivity scenarios at the end of COVID-19, the Montenegrin economy starts to improve and reaches − 1.9%. Still, it seems the macroeconomic resources are not strong enough to keep up the performance, and at the end of December 2020, the output level averages − 3.2%. That is, the Montenegrin economy seems to be vulnerable to external shocks, as it relies heavily upon capital inflows and international tourism to stimulate its growth.

The World Bank forecasted Montenegrin GDP real growth rate at − 5.6% (World Bank 2020a, b), European Commission at − 5.9% (EC 2020), European Bank for Reconstruction and Development at − 8% (EBRD 2020), and International Monetary Fund at − 9% (IMF 2020a, b). Scenario 3 fits between the World Bank, EC, and EBRD, IMF projections.

International tourism is currently not certain considering the fluid pandemic situation. Tourism in Montenegro will depend mostly on the course of new infections, containment measures (primarily neighborhood), medical treatments, and the global disposable income dent. Macroeconomic policymakers should study travel preferences because they could change after the pandemic. Tourism from the Western Balkan neighboring countries constitutes 40% of total tourism, and it seems most likely that tourists will not be able to access Montenegro by air, water, or land.

The spike in health expenditures along with lost tourism revenues can stretch public finances. On March 23, 2020, the Government of Montenegro adopted the first set of measures aimed at facilitating the living standard of citizens and assisting the economy during the coronavirus pandemic. The focus of well-targeted economic and social measures is to keep the current level of employment (data from February 2020) and to protect the most vulnerable categories of the population. The measures included the following: 3-month postponement of repayment of loans given by commercial banks to companies and citizens (in total 3 billion euros), 3-month postponement of payment of taxes and contributions on salaries for all companies and entrepreneurs based on requests to the Tax Directorate, one-off financial assistance to pensioners with the lowest pensions and the beneficiaries of social assistance (1 million euros for 20,000 users), and numerous reductions in budget expenditures (restriction and control of budget spending). The state-owned Investment Development Fund offered a new credit line intended to improve the liquidity of entrepreneurs, SMEs, and large enterprises (120 million euros up to a maximum amount of 3 million euros per beneficiary by a simplified procedure with no approval fee and an interest rate of only 1.5%). These funds are intended for companies operating in the fields of procurement of medicine, medical equipment, and vehicles; tourism and catering; traffic; services; and food production and processing. The second package was presented on April 20, 2020 and consisted of subventions to companies for 2-month minimum gross salaries for registered workers in affected economic sectors and an additional packaging of the budget for agricultural support to farmers (Government of Montenegro 2020).

Based on the previous Montenegrin successful management of revenues and expenditures, the central policymakers should aim to carefully manage the fiscal basket after the recovery of the economy and lower the public debt. Based on the BVARX Litterman/Minessota prior forecasting performance, the central coordinating policymakers should increase health expenditures, as their impact is positive to the output level. Border control should be effective and efficient in testing for COVID-19 and possess the necessary medical resources for this purpose.

## Conclusion and implications

In light of the struggle policymakers are facing owing to COVID-19, to help construct proper policy measures and diagnose the onset of indicators, we identify an approach and methodology that the Government of Montenegro can use for developing the anti-pandemic and overall development strategy. With the increase in the interest in forecasting the cost of growth loss and the lack of a uniform methodology, we believe that the findings presented in our paper will appeal to macroeconomic policymakers. Even though some research papers have identified a few methods that could be used in forecasting the pandemic’s effect on the economy, the methodologies developed from those findings have restrictions and are difficult to administer on a national level. Our results will allow policymakers to understand the variables involved in identifying the onset of COVID-19 impacts and their dynamics and subsequently develop more efficient and effective policy measures that can be used nationally.

The government’s response to the current crisis has been efficient, well-timed, prompt, and largely targeted toward both the economy and socially marginalized categories. The support measures package was well-targeted and in line with the current financial capacity of the budget. The third package was announced in May 2020 and included the budget rebalance measure (raising of the public debt equally to defined additional support measures). The level of public debt could exceed 90% of the GDP. However, these measures, which are focused on securing employment and keeping highly qualified staff in Montenegro’s companies, are justified public finance spending. It will strongly support a new wave of investment in the period after COVID-19.

This study reveals a wide knowledge gap, both theoretical and empirical. We identified a SVAR model recursively. The model aggregates critical macroeconomic variables to forecast the GDP growth rate. Bayesian VARX of Litterman/Minnesota prior has the lowest RMSE compared to the standard VAR and other BVAR priors. We employ the BVAR model to predict all variables under five deterministic dynamic scenarios simultaneously. We also combine and average the forecasts under different scenarios. The evidence shows that human capital, tourism, and health expenditures are critical for sustainable growth. Preserving human capital and not letting exit the country or the economy would amount to significant support and a vital stimulus. Policymakers will need to closely monitor the stock market, as foreign investment will be very fragile after the pandemic has subsided.

In conclusion, a mixture of fiscal and unconventional monetary policies and their implementation in the upcoming months are crucial for sustainable development in Montenegro. The empirical findings of this study provide macroeconomic policymakers with an in-depth understanding of the pandemic’s effects on the Montenegrin economy.

Future research avenues might include sign restrictions and factor-augmented VARX approaches to get a better macro-econometric picture of the COVID-19 outbreak.

## Data Availability

The datasets generated and/or analysed during the current study are available in the Springer Nature Research Data [https://springernature.figshare.com/].

## Appendix

See Fig. 5 and Table 2.

**Table 2:** VAR (2) residual serial correlation LM. *Source*: Authors' calculations

Null hypothesis: No serial correlation at lag h						
Lag	LRE* stat	df	Prob	Rao F-stat	df	Prob
1	47.06455	49	0.5519	0.960131	(49, 639.0)	0.5528
2	11.81389	49	1.0000	0.234634	(49, 639.0)	1.0000
3	5.783233	49	1.0000	0.114337	(49, 639.0)	1.0000
4	10.22446	49	1.0000	0.202822	(49, 639.0)	1.0000
